# Bees may drive the reproduction of four sympatric cacti in a vanishing coastal mediterranean-type ecosystem

**DOI:** 10.7717/peerj.7865

**Published:** 2019-10-07

**Authors:** Pablo C. Guerrero, Claudia A. Antinao, Beatriz Vergara-Meriño, Cristian A. Villagra, Gastón O. Carvallo

**Affiliations:** 1 Departamento de Botánica, Facultad de Ciencias Naturales y Oceanográficas, Universidad de Concepción, Concepción, Chile; 2 Instituto de Entomología, Universidad Metropolitana de Ciencias de la Educación, Santiago, Chile; 3 Instituto de Biología, Facultad de Ciencias, Pontificia Universidad Católica de Valparaíso, Valparaíso, Chile

**Keywords:** Cactaceae, Endemism, Insect pollinators, Plant-animal interactions, Pollen limitation, Hummingbird, Central Chile, Los Molles, Pichidangui, anthropocene

## Abstract

**Background:**

Sympatric congeneric plants might share pollinators, or each species might avoid competition by evolving specialized traits that generate partitions in pollinator assemblages. In both cases, pollen limitation (a decrease in the quality and quantity of compatible reproductive pollen) can occur, driving the plant mating system to autogamy as a mechanism of reproductive assurance. We assessed the relationships between pollinator assemblages and mating systems in a group of sympatric congeneric plants. We attempted to answer the following questions: (i) How similar are pollinator assemblages among sympatric cactus species? (ii) Which mating systems do sympatric cactus species use?

**Methods:**

We studied sympatric *Eriosyce* taxa that inhabit a threatened coastal strip in a mediterranean-type ecosystem in central Chile. We performed field observations on four taxa and characterized pollinators during the years 2016 and 2017. We estimated differences in the pollinator assemblages using the Bray–Curtis index. To elucidate the mating systems, we conducted hand-pollination experiments using three treatments: manual cross-pollination, automatic self-pollination, and control (unmanipulated individuals). We tested differences in seed production for statistical significance using Kruskal–Wallis analysis.

**Results:**

*Eriosyce subgibbosa* showed a distinctive pollinator assemblage among the sympatric species that we studied (similarity ranged from 0% to 8%); it was visited by small bees and was the only species that was visited by the giant hummingbird *Patagona gigas*. Pollinator assemblages were similar between *E. chilensis* (year 2016 = 4 species; 2017 = 8) and *E. chilensis* var. *albidiflora* (2016 = 7; 2017 = 4); however, those of *E. curvispina* var. *mutabilis* (2016 = 7; 2017 = 6) were less similar to those of the aforementioned species. *E. curvispina* var. *mutabilis* showed the highest interannual variation in its pollinator assemblage (18% similarity). Reproduction in *E. subgibbosa* largely depends on pollinators, although it showed some degree of autogamy. Autonomous pollination was unfeasible in *E. chilensis*, which depended on flower visitors for its reproductive success. Both *E. chilensis* var. *albidiflora* and *E. curvispina* var. *mutabilis* showed some degree of autogamy.

**Discussion:**

We observed differences in pollinator assemblages between *E. subgibbosa* and the remaining *Eriosyce* taxa, which depend on hymenopterans for pollen transfer. Pollinator assemblages showed considerable interannual variation, especially those of *E. subgibbosa* (ornithophilous syndrome) and *E. curvispina* var. *mutabilis* (melitophilous syndrome). Autogamous reproduction in these taxa may act as a reproductive assurance mechanism when pollinator availability is unpredictable. Our study contributes to improving our understanding of the reproductive systems of ecological interactions between threatened species in a Chilean mediterranean-type ecosystem.

## Introduction

Pollinator assemblages are a crucial component of plant reproduction (Eckhart, 1992; Johnson, 2010), driving the evolution of plant mating systems (Barrett, 1998; Kalisz, Vogler & Hanley, 2004; Goodwillie, Kalisz & Eckert, 2005). Different factors, such as the taxonomic and functional composition of pollinator assemblages, pollination rates, and pollen flows, influence the ecological dynamics of plant-pollinator interactions, showing significant variation in spatial and temporal dimensions (Johnson & Steiner, 2000; Herlihy & Eckert, 2005; Weber, 2017). Pollinator assemblage properties in sympatric species could occur in a continuum between the two extremes: sharing a wide range of floral visitors (Marques et al., 2007; Schlüter et al., 2009; Ferreira et al., 2018) or specializing in the use of floral visitors, which reduces long-term competition by pollinators (Stone, Willmer & Rowe, 1988). Ultimately, these scenarios can favor pollen limitation either by deleterious impacts of heterospecific pollen transference, such as microallelopathic effects at the stigma level and clogging (Van Der Niet, Johnson & Linder, 2006; Bodbyl Roels & Kelly, 2011; Barrett & Harder, 2017), or by changeable pollination environments (Eckert & Herlihy, 2004; Kalisz, Vogler & Hanley, 2004; Verdú et al., 2006). Extreme scenarios, such as those mentioned above, decrease pollination quality and quantity which can promote rapid evolutionary changes toward selfing and genetic self-compatibility (Kalisz & Vogler, 2003; Moeller & Geber, 2005; Morgan, Wilson & Knight, 2005).

Partitioning pollinator assemblages (specialization or the use of a restricted number of taxa or functional groups; Johnson & Steiner, 2000) can be considered an advantageous strategy when closely related taxa occur in sympatry (Stone, Willmer & Rowe, 1988), which could reduce heterospecific pollen transference (Kudo & Kasagi, 2005). However, adapting specialized pollinator assemblages may increase plant susceptibility to pollen limitation since pollinators show unpredictable richness and density over time (Johnson & Steiner, 2000). In this scenario, the evolution of selfing strategies may be favored in plants (Fenster et al., 2004; Kalisz, Vogler & Hanley, 2004; Kudo & Kasagi, 2005; Knight et al., 2005; Pérez, Arroyo & Armesto, 2009). Experimental evidence has shown a rapid evolution of the pollination system in angiosperms (Kearns, Inouye & Waser, 1998). Indeed, understanding the evolutionary plasticity of pollination systems may contribute to buffering anthropogenic impacts, such as species loss or land use changes, that affect plant-pollinator interactions (Hadley & Betts, 2009; Young et al., 2016).

Among angiosperms, the species of the Cactaceae family have one of the most impressive evolutionary labile reproductive systems (Mandujano et al., 2010; Schlumpberger & Renner, 2012; Hernández-Hernández et al., 2014; Guerrero et al., 2019a). Cacti have evolved conspicuous flowers that attract a wide range of animal pollinators, including vertebrates (e.g., bats, hummingbirds, passerine birds, and lizards) and insects, such as moths, flies, and bees (Mandujano et al., 2010; Guerrero et al., 2012). Cacti exhibit geographical covariation between pollinator assemblages and floral morphology (Schlumpberger et al., 2009; Walter, 2010), a relationship that may reinforce the dependence on biotic pollinators to achieve reproductive success (Valiente-Banuet et al., 1997; McIntosh, 2002; Walter, 2008) and explain the high levels of self-incompatibility exhibited in this family (Boyle, 1997; Martínez-Peralta, Márquez-Guzmán & Mandujano, 2014). Self-compatibility and autonomous systems for reproductive assurance in cacti have been observed in cases of pollinator inconstancy (Nassar & Ramírez, 2004).

Cacti exhibit rich variation in reproductive strategies, although, astonishingly, the reproductive system has been studied in only 2% of species in this family (Mandujano et al., 2010). In addition, most studies of cactus pollination have mainly focused on a single plant species (Mandujano et al., 2010; Larrea-Alcázar & López, 2011; Eggli & Giorgetta, 2015), ignoring the pollination mechanisms of sympatric species (Fleming, Tuttle & Horner, 1996; Eggli & Giorgetta, 2017; Ferreira et al., 2018). Therefore, increasing our knowledge of pollination in sympatric cactus species allows us to identify potential sources of pollen limitation, assess reproductive isolation barriers, and determine mechanisms of seed production. These issues are relevant to identifying demographic bottlenecks in taxa, especially those with high extinction risks. As a way of contributing to filling these gaps in our knowledge of reproductive systems in sympatric plants, we present a study that included four cactus taxa belonging to the *Eriosyce* sect. *Neoporteria* that inhabit a highly threatened ecosystem in coastal central Chile. Specifically, our study attempted to answer the following key questions: (i) How similar are pollinator assemblages among sympatric cactus species? (ii) Which mating systems do sympatric cactus species use?

**Figure 1:**
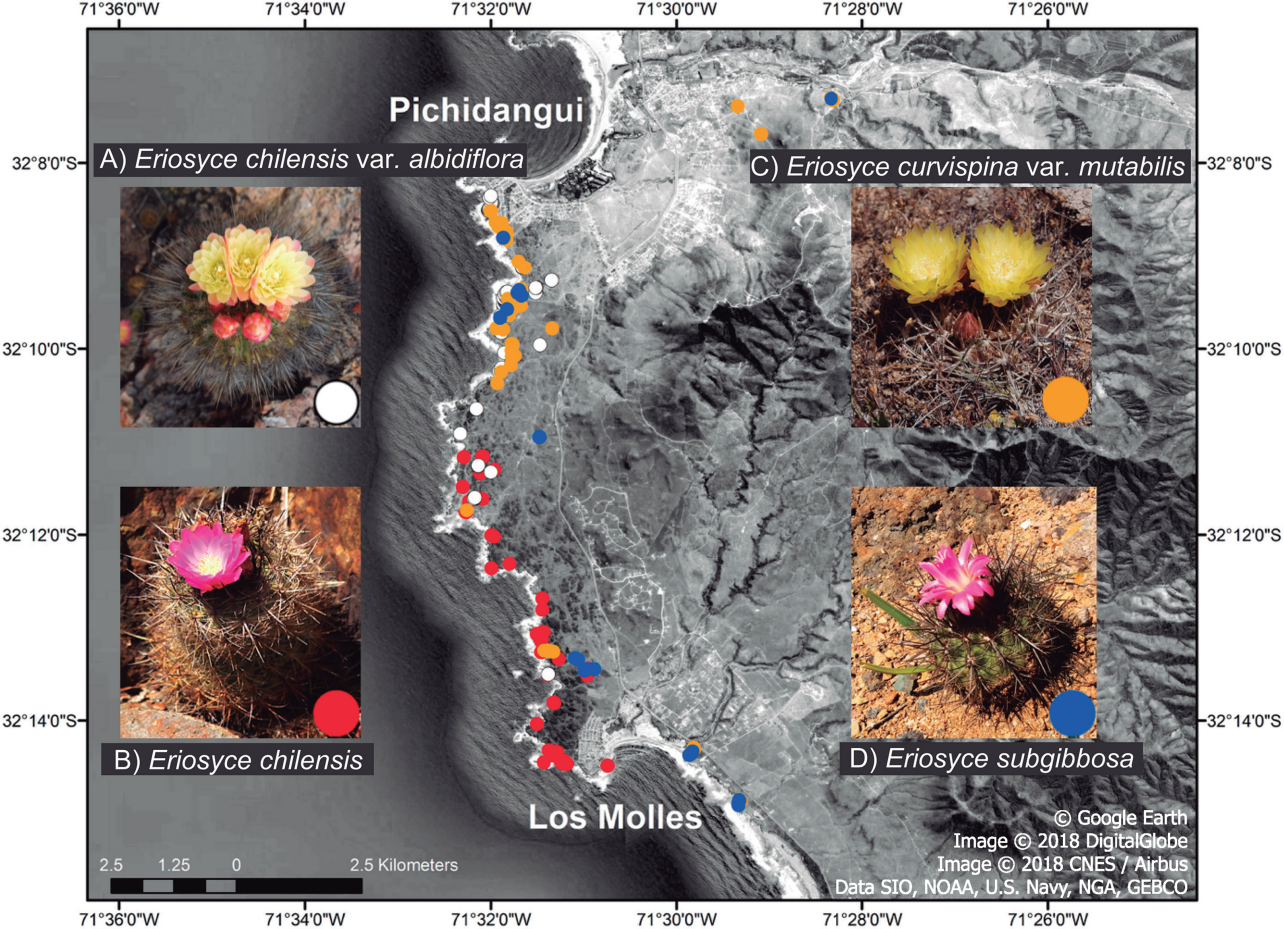
Local distribution of the *Eriosyce* species between Pichidangui Bay and Los Molles Peninsula, Chile. The colors of the dots on the map and within the insets refer to different taxa: (A) *E. chilensis* var. *albidiflora* (white), (B) *E. chilensis* (red), (C) *E. curvispina* var. *mutabilis* (orange) and (D) *E. subgibbosa* (blue). Map data © 2018 Google, Image DigitalGlobe, CNES / Airbus, Data SIO, NOAA, US Navy, NGA, GEBCO.

Since flower morphology potentially predicts the group of pollinators that visit plants (Fenster, Martén-Rodríguez & Schemske, 2009), we expect that *Eriosyce* taxa with funnel-shaped flowers (i.e., *Eriosyce chilensis*, *E. chilensis* var. *albidiflora* and *E. curvispina* var. *mutabilis*; Fig. 1) will be bee pollinated sharing pollinators. In contrast, species that have internal tepals curved inwards forming tubular flowers (i.e., *E. subgibbosa*, Fig. 1), a trait previously suggested as an adaptation to an ornithophilous pollination syndrome (Walter, 2008), may be hummingbird pollinated and thus should present substantial differences in its pollinator assemblage compared to the remaining taxa. We also expect some degree of self-compatibility in all taxa, as previously suggested in a previous study (Walter, 2008).

## Materials and Methods

### Study site

We carried out our study in a coastal strip of central Chile between Pichidangui Bay (32°08′S; 71°32′W) and Los Molles Peninsula (32°14′S; 71°30′W) (Fig. 1). This area is the most significant remnant of coastal Mediterranean-type scrubland; it is dominated by sclerophyllous vegetation (Luebert & Pliscoff, 2006; Alaniz, Galleguillos & Perez-Quezada, 2016) that is characterized by its high plant diversity, with 57% consisting of flowering species endemic to central Chile (Lund & Teillier, 2012). Today, 17 plant taxa from this area are in a threatened status (Lund & Teillier, 2012) due to anthropogenic pressures. The study was conducted in a publicly accessible area (no permit required).

### Study species

We studied all species of the genus *Eriosyce* that are present in this area, which are sympatrically distributed with partial or full overlaps in their ranges (Fig. 1; Guerrero et al., 2011): *E. chilensis* (Hildm. ex K. Schum.) Katt., *E. chilensis* var. *albidiflora* (F. Ritter) Katt., *E. subgibbosa* (Haw.) Katt., and *E. curvispina* (Bertero ex Colla) Katt. var. *mutabilis* (F. Ritter) Katt. The first three species belong to the *Neoporteria* section, while the fourth species belongs to the *Horridocactus* section (Guerrero et al., 2019b). Two of these taxa (*E. chilensis* and *E. chilensis* var. *albidiflora*) are narrow endemics that inhabit exclusively studied areas, and many other species in the family have reduced their potential distribution range through the effects of habitat loss in recent decades (Goettsch et al., 2015). This situation has led to *E. chilensis* being classified as critically endangered (Faundez et al., 2013). Despite having wide distributional ranges, *E. subgibbosa* and *E. curvispina* var. *mutabilis* only have an overlapping distribution in the studied site (Fig. 1). *Eriosyce chilensis* is a globose cactus that exclusively inhabits rocky outcrops near the coast. This taxon has two taxonomic varieties. The nominal species *E. chilensis* has flowers with pink tepals and occurs in all studied areas except in the northernmost portion (near Pichidangui) (Fig. 1). The second variety is *E. chilensis* var. *albidiflora*, a local endemic that is distributed in the northernmost range of the species and has flowers with white tepals. Both taxonomic varieties of *E. chilensis* have flowers with tepals deflected outwards (funnel-shaped flowers; Fig. 1). *Eriosyce subgibbosa* is a globular-elongated cactus that is distributed on the Pacific coastline in a 600 km range between 31°S and 37°S and presents pink to pale pink tepals that form a narrow tube with the internal tepals curved inwards (tubular flowers; Fig. 1). These taxa occur in the northern and southern ranges of the study site and occupy coastal rocky outcrops and cliffs (Walter, 2008). *Eriosyce chilensis*, *E. chilensis* var. *albidiflora* and *E. subgibbosa* are very similar in their vegetative structures but display more substantial differences in flower morphology (Fig. 1). *Eriosyce subgibbosa* has a rather tubular-shaped hypanthium in comparison with the funnel-shaped flowers of *E. chilensis* and *E. chilensis* var. *albidiflora*. Besides, the latter two species differ in the pigmentation of their flowers, as *E. chilensis* can be seen as pink by human vision, while *E. chilensis* var. *albidiflora* is white and yellowish. Details of diagnostic characters for the determination of Chilean *Eriosyce* are available in Kattermann (1994). Finally, *Eriosyce curvispina* var. *mutabilis* is a globular cactus that is narrowly distributed from 32°S to 32°30′S in coastal terraces and valleys with oceanic influence. This species has a hemicryptic habit, living semi-buried (Kattermann, 1994). This species has showy flowers with outward tepals (similar to those of *E. chilensis*) that are pigmented yellow to orange (Fig. 1). The floral morphology of *E. curvispina* var. *mutabilis* suggest that this species is bee-pollinated.

### Visitors to *Eriosyce* flowers

We performed field observations to determine the main pollinators of each *Eriosyce* taxon during its peak period of flower anthesis: August (*E. subgibbosa*), October (*E. chilensis* and *E. chilensis* var. *albidiflora*) and November (*E. curvispina* var. *mutabilis*). Despite differences in peak flowering times among the species, there is some overlap between individuals in their floral phenology from August to December. Observations for each taxon were performed during two consecutive years (2016 and 2017) in 25 m^2^ plots that contained clumps of 2–17 adult individuals (12.7 ± 1.5 individuals per plot, mean ± standard error) with 2.5 ± 0.5 flowers per individual. For each species, the plots were at least 20 m away from each other. We conducted observations on three consecutive days between 8:00 and 20:00 h in periods of 30 min per plot. Four observers were assigned to each species except for *E. curvispina* var. *mutabilis*, for which we had two observers. Observation times coincided with the flower anthesis of each species; once opened, flowers remained in this state. Previous observations after 20:00 h led us to disregard the presence of nocturnal visitors. This methodology allowed us to cover 540 min of observation each day per observer. We performed a total of 6,480 min of observation per species (540 min × 4 observers × 3 consecutive days), except for *E. curvispina* var. *mutabilis*, for which we accumulated 3,240 min of observation.

When animals approached the flowers, we recorded the time they entered the flowers and collected the animals when feasible. Since we do not evaluate the effectiveness of animal visitors as pollinators, we considered all animals that entered the flowers and contacted reproductive parts (stigmas and anthers) to be pollinators. The collected specimens were pin-mounted and taken to the laboratory for later identification by Apoidea expert Dr. Luisa Ruz (Pontifical Catholic University of Valparaiso, Chile). Brochure specimens were deposited in the Entomology Collection of the University of Concepción (MZUC-UCCC), Chile.

Based on flower visits, we built a plant-pollinator matrix for each year. We estimated pollinator richness using matrix randomization (*N* = 9,999 randomizations) based on models that find 2 × 2 submatrices that can be swapped while holding the totals of the columns (pollinators) and rows (plant individuals) constant (“swap count” algorithm, Gotelli & Entsminger, 2001; Gotelli & Entsminger, 2003). Additionally, we estimated the similarity of pollinator assemblages among *Eriosyce* cacti, both between years within each taxon and among taxa within each year, using the Bray–Curtis dissimilarity index (Bray & Curtis, 1957), this index ranges between 0 (two samples share all species) and 1 (two sites do not share any species). Since the Bray–Curtis index (hereafter *BC*) is a dissimilarity index, we reported similarity as 1—*BC*. All these analyses were performed using the library *vegan* (Oksanen et al., 2018) in R Software version 3.5.0 (R Development Core Team, 2018).

### Dependence from pollinators and mating system

We marked and bagged flower buds of each cactus species during its bloom peak: *E. subgibbosa* in August 2017 (*N* = 70 individuals); *E. chilensis* var. *albidiflora* (*N* = 32 individuals) and *E. chilensis* (*N* = 55 individuals) in October 2016; and *E. curvispina* var. *mutabilis* (*N* = 75 individuals) in November 2017. We used plastic silk fabric (200 × 200 mm) to bag the flowers, which prevented animals from entering the flowers.

We monitored individuals daily, and as anthesis proceeded, we randomly assigned one of the following treatments to each individual: (i) manual cross-pollination, in which the flowers were pollinated with pollen from individuals located at least five m away from the focal individuals; (ii) automatic self-pollination, in which the flowers were kept bagged; and (iii) the control, in which the bags were removed on the day of anthesis and the flowers were unmanipulated (following Eckert et al., 2010). For manual cross-pollination, the donor individuals were 2-day-old flowers. Preliminary assays and the results of this study attest that this pollen is viable. Only one flower was treated per focal plant because the pollination of more than one flower may lead to resource limitations (Charnov, 1979), which may cause seed production to vary among individuals with a different number of treated flowers.

Before performing pollination experiments, we assessed the receptivity of stigma by using the bubble production method, which involves immersing the stigmas in a 3% hydrogen peroxide solution and observing bubble production for 2–3 min. This bubbling indicates the activity of stigmatic peroxidases and, hence, the receptivity of the stigma (Armbruster et al., 2002). The receptivity of stigmas was assayed in 1- to 2-day-old flowers (*N* = 10 flowers from different individuals of each species), and we observed receptivity in 100% of the flowers. All taxa of *Eriosyce* included in this study showed stigma receptivity even before anthesis (we assessed the receptivity of some stigmas in floral buds). We collected tagged fruits for counting seeds four weeks after applying the treatments.

We assessed differences in seed number among treatments for each plant species using a Kruskal–Wallis analysis (Sokal & Rohlf, 1995). When we found significant differences, we performed Nemenyi a posteriori tests (Zar, 1996).

## Results

*Eriosyce* flowers were visited by 16 taxa during two consecutive years; 199 and 303 visits were observed in 2016 and 2017, respectively (Table 1; Appendix 1). In general, after blooming, *Eriosyce* flowers remained open for a period of 4–6 days. The *Eriosyce* species did not differ statistically in their pollinator richness (Table 1), and the similarity of pollinator assemblages was relatively low between year for each taxon (<0.5 of similarity; Table 2). Floral pollinators of *Eriosyce* belonged to two main groups: bees of the superfamily Apoidea (Hymenoptera) and the giant hummingbird *Patagona gigas* (Trochilidae: Apodiformes; Appendix 1). All observed bees were native species (*N* = 14) except for *Apis mellifera* Linnaeus, 1758, with three visits accounted for only 0.9% of visits to *E. chilensis* var. *albidiflora* (Appendix 1).

**Table 1:** Pollinator richness and number of visits for four *Eriosyce* taxa in two consecutive years, central Chile.

	Year 2016		Year 2017	
Taxon	Species richness	Number of visits	Species richness	Number of visits
*E. chilensis*	4 (3–8)	32	8 (4–10)	74
*E. chilensis* var. *albidiflora*	7 (3–9)	32	4 (4–10)	97
*E. curvispina* var. *mutabilis*	7 (4–11)	85	6 (4–10)	118
*E. subgibbosa*	4 (3–9)	50	2 (2–7)	14
Total	11 (4–10)	199	12 (5–11)	303

**Note:**

Values in parentheses in species richness are 95% confidence intervals estimated after matrix randomization (*N* = 9,999).

**Table 2:** Similarity of pollinator assemblages (1—Bray–Curtis index) among the *Eriosyce* species during 2016 (below the diagonal) and 2017 (above the diagonal). The diagonal depicts intraspecific similarity between the years 2016 and 2017. The observed similarity indexes were contrasted with null models to test whether they were different from the expected by chance, values that deviated from null expectations are marked with asterisks (*P < 0.05), the interval of null models are shown in parenthesis.

	*E. chilensis* var. *albidiflora*	*E. chilensis*	*E. curvispina* var. *mutabilis*	*E. subgibbosa*
*E. chilensis* var. *albidiflora*	0.403 (0.050–0.851)	0.690 (0.070–0.837)	0.242 (0.027–0.857)	0.018 (0–0.851)
*E. chilensis*	0.844 (0.051–0.847)	0.453 (0.073–0.833)	0.104* (0.154–0.860)	0 (0–0.718)
*E. curvispina* var. *mutabilis*	0.291 (0.165–0.868)	0.342 (0.049–0.843)	0.187 (0.136–0.872)	0.030 (0–0.776)
*E. subgibbosa*	0.032* (0.064–0.833)	0 (0–0.862)	0.089 (0.050–0.852)	0.438 (0–0.811)

*Eriosyce subgibbosa* showed the most distinctive pollinator assemblage compared to the remaining taxa (Table 2; Appendix 1), which could be attributed to visits from *P. gigas*, which exclusively visited this cactus species. Pollinator assemblage of *E. subgibbosa* was similar to the other *Eriosyce* species in 0% to 8.9% (year 2016) and 0% to 3.1% (year 2017; Table 2). Contribution of *Patagona gigas* to the total visits received by *E. subgibbosa* was 80% (2016) and 85% (2017). Remaining visits to *E. subgibbosa* were carried out by native bees (Appendix 1). The three other *Eriosyce* species, which have funnel-shaped floral morphology, showed more similar floral assemblages within them, and hummingbird visits were not observed (Table 2). The most similar pollinator assemblages were observed between *E. chilensis* and *E. chilensis* var. *albidiflora* (84.4% and 69.1% for 2016 and 2017, respectively). *Eriosyce curvispina* var. *mutabilis* showed a distinctive pollinator assemblage compared with the other funnel-shaped species and exhibited the highest interannual variation in pollinator composition among all *Eriosyce* taxa (Table 2).

**Figure 2:**
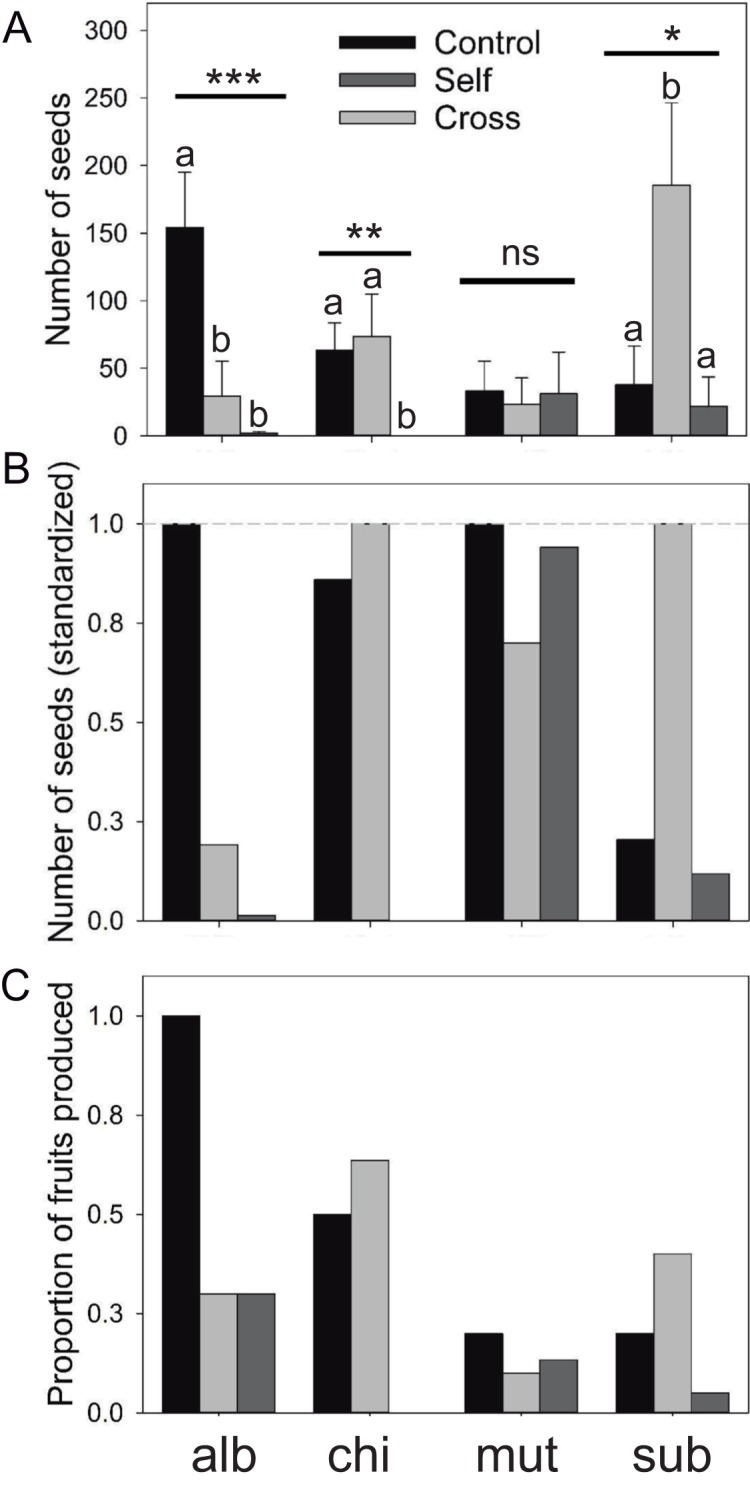
Reproductive output of the *Eriosyce* species after the pollination experiment. (A) Number of seeds (mean ± standard error), (B) number of seed (standardized) and (C) proportion of fruits produced. For each panel, the bars indicate the results of the three pollination treatments: unmanipulated plants (Control), manually cross-pollinated plants (Cross) and automatically self-pollinated plants (Self). Taxa names were abbreviated as follows: *E. chilensis* var. *albidiflora* (alb), *E. chilensis* (chi), *E. curvispina* var. *mutabilis* (mut) and *E. subgibbosa* (sub). For seed production, we reported the statistical significance of the Kruskal–Wallis test that compared differences among treatments for each species (***P < 0.001; **P < 0.01; *P < 0.05; ns, non-significant differences). Letters above seed number bars are the results of Nemenyi a posteriori tests; different letters indicate significant differences among compared treatments.

Results of the mating treatment in *E. subgibbosa* indicate significant pollen limitation since manually cross-pollinated flowers produced more seeds than unmanipulated flowers (Kruskal–Wallis χ^2^ = 6.987; *P* < 0.031; Fig. 2). Although it was not an overdispersed phenomenon, only one *E. subgibbosa* individual generated seeds under the automatic self-pollination treatment; thus, autonomous selfing cannot be excluded as a reproductive system in this species (Fig. 2). *Eriosyce* species with funnel-shaped flowers showed differences among their mating systems. *Eriosyce curvispina* var. *mutabilis* supported autonomous self-pollination and no differences in seed production were observed among compared treatments (χ^2^ = 0.637; *P* = 0.727; Fig. 2). *Eriosyce chilensis* showed allogamous pollination, which may be largely dependent on pollinators (χ^2^ = 9.91; *P* < 0.001; Fig. 2). In *E. chilensis* var. *albidiflora*, autonomous pollination had a marginal contribution, whereas allogamous pollination was potentially more important to seed production (Fig. 2; χ^2^ = 16.83; *P* < 0.001).

## Discussion

We found contrasting pollinator assemblages among sympatric *Eriosyce* species; they exhibited floral morphology associated with the ornithophilous syndrome (*E. subgibbosa*) and the melitophilous syndrome (the remaining taxa). Interestingly, *E. subgibbosa* was the only species that interacted with the giant hummingbird (*Patagona gigas*), although it also received visits from some relatively small native bees (e.g., *Dialictus* species from Halictidae family were their most frequent visitors). As found in other studies in cacti, plants with ornithophilous syndromes do not necessarily restrict other pollinators from using the resources that they offer (Gorostiague & Ortega-Baes, 2016). The similarity of pollinator assemblages in species funnel-shaped flowers was not homogeneous since *E. chilensis* and *E. chilensis* var. *albidiflora* present the higher similarity while *E. curvispina* var. *mutabilis* was the most dissimilar funnel-shaped species.

A substantial interannual variation in pollinator assemblage was observed, with more than three times fewer total visits (hummingbirds and hymenopterans) to *E. subgibbosa* during the 2nd year of our study. The presence of other plants with massive nectar production may reduce visits to *E. subgibbosa*, which produces less nectar compared to species of the genus *Puya* that are abundant in the study site (Walter, 2008; Hornung-Leoni, González-Gómez & Troncoso, 2013). Plants that produce massive amounts of nectar interfere in the pollination process of plants with less nectar production (Fleming et al., 2001; Chittka & Schürkens, 2001). For instance, *Puya chilensis* Mol. and *P. venusta* Phil. ex Baker. co-occur in the study site and exhibited a massive bloom during the year 2017 (P Guerrero, 2017, personal observations), suggesting a pollination interference against *E. subgibbosa*, because this last species have smaller nectar chambers and therefore produce less nectar (Walter, 2008). In addition, changes in pollinator frequency and diversity may promote reproductive constraints in *E. subgibbosa* during certain years. Indeed, we detected pollen limitation mediated by pollinators since manually cross-pollinated flowers produced a higher number of seeds than unmanipulated flowers. Pollen limitation is a widely represented phenomenon in angiosperms (Bennett et al., 2018), and it acts as a significant evolutionary driver since pollen limitation is a selection agent for mating systems (Totland, 2001). The pollination scenario in *E. subgibbosa* indicates a specialized and interannually erratic assemblage, suggesting a pollen limitation that could be hampered by the existence of selfing, which has been observed in other cactus species (Nassar & Ramírez, 2004). Also, together with natural interannual variation in pollinator visit frequencies, anthropogenic disturbances that occur at a site (e.g., changes in land use, desertification) may negatively impact pollinator visit frequencies in locally endemic cactus (Strong & Williamson, 2007; Martínez-Peralta & Mandujano, 2016). Our results support facultative selfing in *E. subgibbosa*, which may act as a reproductive assurance mechanism that may become increasingly important as the number of ***Patagona* gigas** visits decreases.

In our study, we found that *Eriosyce* species with funnel-shaped flowers were strictly bee-pollinated, which is consistent with their morphology and flowering peaks, which occur during mid-spring when the increasing day temperature drives increasing pollinator activity. Although the diversity of native bees in the xeric areas of Chile is relatively low compared to other mediterranean ecosystems (Michener, 1979), native bees are a crucial component of the pollination process (Montalva & Ruz, 2010; Medel, González-Browne & Fontúrbel, 2018). Previous studies on the same bees species that visited *Eriosyce* showed that they are generalists in the use of floral resources (Montalva & Ruz, 2010; Michener, 2007), such as *Alloscirtetica lanosa* (Apidae), *Diadasia chilensis* (Apidae), *Xenochilicola diminuta* (Colletidae) and *Caenohalictus cyanopygus* (Halictidae). Similarly, we observed a generalist behavior of bees that visited the studied species; this may favor heterospecific pollen transfer among taxa and may open a window of ecological opportunity for hybrid formation. Compared to the giant hummingbird, insects have been less studied, and thus ranges of Chilean bees are far from being understood. This state of the art is related to the fact that most of the distribution ranges of Chilean bees are only reported at latitudinal level (Montalva & Ruz, 2010). In addition, several Apoidea pollinators observed in this study seem to correspond to new native bee species (i.e., *Anthrenoides* sp. 1, *Liphanthus sp*. 1, L Ruz, 2018, personal communications.) whose distributional ranges and ecological associations have not yet been described. In this sense, the distribution, local abundances, and foraging preferences of the observed bees are topics that require more attention. Otherwise, the role of native bee species in plant reproduction in central Chile will remain deficient.

Bee-pollinated *Eriosyce* species, which consistently displayed funnel-shaped flowers, exhibited variation on pollinator dependence for their reproductive success. Although self-incompatibility has been reported in at least 31 genera of cacti (Boyle, 1997), including some *Copiapoa* and *Eriosyce* species (Ganders, 1976). We observed some degree of autonomous reproduction in *E. chilensis* var. *albidiflora* and *E. curvispina* var. *mutabilis*, this was expected since a previous study highlighted that species of the *Eriosyce* subsection *Neoporteria* have some degree of self-compatibility (Walter, 2008). Our result is coherent with a recent meta-analysis that showed that mixed mating is frequent among angiosperms (Whitehead et al., 2018). Also, our study supports the existence of a mixed reproductive system in cacti (i.e., *E. curvispina* var. *mutabilis*), that may act as reproductive backup under erratic pollinator environments. It is interesting to observe that funnel-shaped flowers that exhibited higher similarity in their pollinator assemblages (*E. chilensis* and *E. chilensis* var. *albidiflora*), both taxa also occupy the same microhabitat-type (i.e., coastal rocky outcrops and cliffs; Guerrero et al., 2011). Microhabitat conditions may be crucial for maintaining pollinator consistency among taxa and across years (Schatz, 2006; Adedoja et al., 2019). *Eriosyce curvispina* var. *mutabilis* that inhabit coastal terraces showed marked differences in their pollinator assemblage and a mixed mating system, supporting both allogamous and autogamous strategies. Coastal terraces are sites with harsh pollination conditions since the open spaces are subject to wind gusts, pollinator predators may be present, and there may be more frequent anthropogenic disturbances (Carvallo et al., 2019). Under this scenario, it is likely that *E. curvispina* var. *mutabilis* evolved a mixed mating system with some degree of autonomous selfing (Eckert et al., 2010). This may allow *E. curvispina* var. *mutabilis* to maintain some level of seed production during pollinator-depauperate seasons.

The observed variation in mating systems among the sympatric *Eriosyce* species that we studied raises scientific questions about how the pollination syndromes evolved, and whether mating system evolution played a role in the evolutionary origin of the narrow endemic *E. chilensis*. This because species belonging to the Neoporteria clade are characterized by an ornithophilous syndrome, but our study revealed strict melitophilous reproduction. This result and the phylogenetic topology of the clade (Guerrero et al., 2019b), suggest the reversal of a plesiomorphic state in the Neoporteria from bird pollination back to bee pollination. Previous studies in the Cactaceae have revealed that changes in pollination systems can increase diversification rates by promoting speciation (Schlumpberger & Renner, 2012; Hernández-Hernández et al., 2014). Finally, our study highlights the necessity of further studies that could test the existence and potential effects of inbreeding depression, particularly in species with some degree of autogamous reproduction. Inbreeding depression must be considered in the context of a disturbed landscape because it can promote population and genetic bottlenecks, especially in narrowly endemic species such as those included in this study.

## Conclusions

We found a close relationship between floral morphology and pollinator assemblages since funnel-shaped flowers were mainly visited by small-sized native bee species and the tubular-shaped flower was visited by the giant hummingbird. The higher similarity in pollinator assemblages within bee pollinated taxa may contribute to the hybrid formation, but to test whether this process is effectively occurring in nature, new analyses based on molecular data must be performed. Also, our study contributes to highlighting the potential role of self-compatibility when pollinators are scarce or unpredictable as a mechanism of reproductive assurance. However, longer observations are required to test this hypothesis, this mechanism could be critical in the long term persistence since the reduction of pollinator abundance may have negative impacts in plant reproduction success. This is an important issue, especially in the cactus family, which is a threatened group at a global scale that includes several taxa with a narrow distribution.

## Supplemental Information

10.7717/peerj.7865/supp-1 Supplemental Information 1 Hummingbird and bees species visiting frequency on flowers of four *Eriosyce* during two consecutive years in coastal central Chile.

10.7717/peerj.7865/supp-2 Supplemental Information 2 Raw data of pollinators, reproductive output and nectar production.

## Additional Information and Declarations

### Field Study Permissions

The following information was supplied relating to field study approvals (i.e., approving body and any reference numbers): No permit required for doing field work on coastal area.

### Data Availability

The following information was supplied regarding data availability: The raw measurements are available in the Supplemental Files.
