# Supplementary material for: Bees may drive the reproduction of four sympatric cacti in a vanishing coastal mediterranean-type ecosystem
Source: PeerJ. 2019 Oct 7;7:e7865. doi: 10.7717/peerj.7865 (PMC6786246; doi:10.7717/peerj.7865)
Supplement: Supplemental Information 1 [file peerj-07-7865-s001.docx]

**Appendix 01**

| Family | Species | Relative frequency | | | | | | | |
| --- | --- | --- | --- | --- | --- | --- | --- | --- | --- |
|  |  | *Eriosyce subgibbosa* | | *Eriosyce curvispina* var. *mutabilis* | | *Eriosyce chilensis* | | *Eriosyce chilensis* var. *albidiflora* | |
|  |  | 2016 | 2017 | 2016 | 2017 | 2016 | 2017 | 2016 | 2017 |
| Trochilidae | *Patagona gigas* Giant hummingbird | 40 (80%) | 12 (85%) |  |  |  |  |  |  |
| Andrenidae | *Anthrenoides* sp. 1 |  |  | 2 (0.2%) | 91 (81%) | 14 (41%) | 6 (0.8%) | 13 (40%) | 25 (26%) |
| Andrenidae | *Liphanthus* sp. 1 |  |  | 30 (35%) |  | 13 (40%) | 52 (70%) | 12 (37%) | 70 (72%) |
| Apidae | *Alloscirtetica lanosa* Urban, 1971 |  |  |  | 3 (0.2%) |  |  |  |  |
| Apidae | *Apis mellifera* Linnaeus 1758 |  |  |  |  |  |  | 3 (0.9%) |  |
| Apidae | *Diadasia chilensis* (Spinola 1851) |  |  | 1 (0.1%) |  |  |  |  |  |
| Colleditadae | *Chilicola mantagua* Toro and Moldenke 1979 |  |  | 8 (0.9%) |  |  |  |  |  |
| Colleditadae | *Chilicola* sp. 1 |  |  | 11 (12%) | 2 (0.1%) | 3 (0.9%) | 9 (12%) | 1 (0.3%) |  |
| Colleditadae | *Xenochilicola diminuta* Toro and Moldenke, 1979 |  |  |  |  |  | 1 (0.1%) |  |  |
| Halictidae | *Caenohalictus cyanopygus* Rojas and Toro, 2000 | 1 (0.2%) |  |  |  |  |  |  |  |
| Halictidae | *Caenohalictus rostraticeps* (Friese, 1917) | 3 (0.6%) |  |  |  |  | 2 (0.2%) | 1 (0.3%) |  |
| Halictidae | *Caenohalictus* sp. 1 |  |  |  | 7 (0.5%) |  |  |  |  |
| Halictidae | *Corynura herbsti* (Alfken, 1913) |  |  |  |  |  | 1 (0.1%) |  |  |
| Halictidae | *Dialictus* sp. 1 | 6 (12%) | 2 (14%) | 27 (31%) | 13 (11%) |  |  | 1 (0.3%) | 1 (0.1%) |
| Halictidae | *Dialictus* sp. 2 |  |  |  |  |  | 1 (0.1%) |  |  |
| Megachilidae | *Trichothurgus dubius* (Sichel, 1867) |  |  | 6 (0.7%) | 2 (0.1%) | 2 (0.6%) | 2 (0.2%) | 1 (0.3%) |  |
|  | Total | 50 | 14 | 85 | 118 | 32 | 74 | 32 | 96 |
